# Physical activity and risk of sudden cardiac death in individuals at high risk for cardiovascular disease: A protocol for systematic review and meta-analysis

**DOI:** 10.1097/MD.0000000000025890

**Published:** 2021-05-14

**Authors:** Qinqin Wu, Fanghui Li, Yu Jia, Yi Liu, Rui Zeng

**Affiliations:** a Health Management Center; b Department of Cardiology; c Department of Emergency Medicine, West China Hospital, Sichuan University, Chengdu, China.

**Keywords:** cardiovascular disease, physical activity, sudden cardiac death

## Abstract

**Background:**

Regular physical exercise is recommended for lowering the risk of coronary events and all-cause mortality. However, there is a variety of exercise options, and their relative effectiveness and hierarchy remain unclear. Herein, we present a systematic review and meta-analysis protocol that aims to compare the impact of different types of physical activity on the risk of sudden cardiac death.

**Methods and analysis:**

English language articles reporting on randomized controlled trials will be searched in MEDLINE, Cochrane Library, CINAHL, and PubMed databases by two reviewers. A snowball approach will be used for literature retrieval. Reviewers will independently screen the literature, extract data, evaluate study quality and risk of bias, and perform a meta-analysis. In the presence of heterogeneity, a random effects model will be used for meta-analysis; otherwise, a fixed effect model will be used.

**Ethics and dissemination:**

This study is a secondary analysis and does not require ethical review. The results of this study will be disseminated through peer-reviewed publications, journals, and academic conferences and other forms of exchange.

**Systematic review registration number:**

INPLASY202140033

## 1 Introduction

Cardiovascular disease (CVD) is the leading cause of death worldwide.^[1]^ Sudden cardiac death (SCD) accounts for 40–50% of CVD cases and 15–20% of all deaths.^[2]^ SCD usually occurs shortly after the onset of non-specific symptoms; given the little time available for an effective intervention, SCD is often fatal. Some studies have shown that^[3–8]^ obesity, smoking, coronary heart disease, and high blood pressure are risk factors for SCD. However, the types of intervention that may prevent SCD remain unclear. Physical activity has been suggested to reduce the risk of SCD, helping reduce the associated public health burden.

In adults, the relationship between physical activity and all-cause mortality, cardiovascular mortality, hypertension, and diabetes is well-documented.^[9,10]^ Physical activity can promote many physiological responses that may translate into short- and long-term autonomic and hemodynamic adaptations. As a result, it may reduce the risk of hypertension, which is a major risk factor for CVD. In fact, some evidence^[11]^ suggests that the amount of physical activity is inversely proportional to the incidence of high blood pressure in adults with normal blood pressure at baseline. In addition, increased levels of physical activity are associated with decreased risk of death. A recent study has shown^[12–14]^ an inverse curvilinear dose-response relationship between the levels of physical activity and CVD-related mortality rate. The World Health Organization Physical Activity and Sedentary Behavior Guidelines recommend^[10]^ that adults should do at least 150–300 min of moderate-intensity aerobic activity or 75–150 min of high-intensity aerobic activity or an even amount of both types of activity per week.

Physical activity is associated with both benefits and risks.^[15–17]^ For example, while some studies have shown that physical activity can reduce the risk of SCD, other studies have shown that it can trigger SCD.^[18–20]^ An estimated 6–17% of SCD cases in men have been related to acute strain. The risk of SCD associated with exertion is lower in women than in men (1 in every 36 million hours).^[4]^ Although exercise-related SCD is relatively rare, it remains a concern for both doctors and patients.

Findings on the link between the levels of physical activity and CVD-related mortality rates have been consistent.^[21]^ However, the dose-response relationship between the amount or/and intensity of physical activity and specific health outcomes remains unclear. The present systematic review and meta-analysis aim to examine the types and intensity of physical activity that are most effective at reducing the risk of SCD and to clarify any dose-response relationship between these variables.

## 2 Methods

The present study will adhere to the Preferred Reporting Items for Systematic Reviews and Meta-Analysis Protocols statement and Cochrane Handbook for Systematic Reviews of Diagnostic Test Accuracy. This protocol for systematic review and meta-analysis has been registered with International Platform of Registered Systematic Review and Meta-analysis Protocols (INPLASY202140033).

### 2.1 Eligibility criteria

Only reports of randomized controlled trials published in the English language at any time are eligible for inclusion in the present study. The aim of eligible trials was to promote exercise among adults (age ≥ 18 years) at high risk of CVD and physically inactive at baseline (moderate exercise for < 30 min/week, 3 times a week, or < 90 min of medium-intensity exercise per week). The Framingham and Systematic Coronary Risk Evaluation score was used to predict CVD mortality risk. Participant exclusion criteria were:

1. history of cardiac shock or cardiac arrest;
2. impaired mobility;
3. diagnosed with CVD; and
4. unstable vital signs. Studies will be included if they provide data on physical activity frequency, intensity, and type and repetition time as part of the intervention.

Other types of research, such as observational studies, animal trials, research protocols, duplicate reports, and ongoing trials will be excluded.

### 2.2 Types of interventions

Studies involving any types of physical activity are eligible, without restrictions on frequency, intensity, or duration of exercise. The types of exercise could include aerobic (low- or medium-intensity continuous exercise and low-, medium-, or high-intensity interval exercise), resistance, flexibility, or a combined form of training. Exercise-based intervention is defined as a planned, cumulative, repetitive exercise behavior for at least 6 weeks, classified based on the World Health Organization guidelines as poor (0 min/week of moderate or vigorous exercise), moderate (1–149 min/week of moderate exercise or 1–74 min/week of vigorous exercise), and recommended (≥ 150 min/week of moderate exercise or ≥ 75 min/week of vigorous exercise). Studies will be excluded if they combine exercise with other health interventions such as nutrition and psychological interventions and where data on physical activity alone cannot be extracted. All intervention settings are eligible for inclusion, for example, outpatient and inpatient, home-based, and community-based rehabilitation. The control group will be the study group not participating in physical activity.

### 2.3 Types of outcome measures

The primary outcomes are mortality due to SCD, CVD-related hospitalization, and re-hospitalization rates. The secondary outcomes are peak oxygen consumption (peak levels of VO_2_), peak work rate (peak WR), VE/VCO_2_ levels, left ventricular ejection fraction (LVEF), 6-minute walking distance (6MWD), health-related quality of life, depression scores, exercise compliance (defined as the number of completed over that of the total prescribed courses), and adverse events related to heart disease (falls, fractures). Health-related quality of life and depression may be evaluated by any of the validated tools, for example, the Barthel index, which evaluates functional dependence of patients in activities of daily living. The Barthel index includes 10 items; the highest total score is of 100 points. The score of 0 points is assigned to patients who fail to meet the project criteria; a score of ≥ 60 points indicates a patient that can live independently; scores of 40–60 points indicate some care needs; scores of 20–40 points indicate high care needs; finally, scores of < 20 points indicate high care needs. Depression may be evaluated with the Hamilton Depression scale (HAMD). The HAMD consists of 21 questions; the scores of 0–7, 7–13, 14–18, 19–22, and > 22 points indicate normal state, and mild, moderate, severe, and extremely severe depression, respectively. Reported compliance of ≥ 75% during a given follow-up period is considered indicative of a successful intervention.

### 2.4 Search strategy

Two reviewers will independently search the MEDLINE, Cochrane Library, CINAHL, and PubMed databases for articles published in the English language at any time. A snowball approach to literature retrieval will be used, including a search of reference lists of the retrieved articles and published comments on the subject. To be eligible, articles must include a detailed description of exercise intervention, specifically, the type, frequency, duration, and intensity of exercise. The duration of intervention should be of ≥ 6 weeks. We will expand the search scope of the database (by searching the OpenGrey database) to identify other relevant studies, including unpublished studies and references in grey literature, as required. Literature retrieval will involve a combination of logical conjunctions such as “OR” and “AND” and a combination of specialist terminology to identify titles and abstracts of potentially relevant articles. Any discrepancies in the process will be resolved by consensus between two reviewers, or arbitration by the third reviewer, as required. Table 1 shows the results of literature retrieval by subject words in PubMed.

**Table 1:** Results of the literature retrieval in PubMed.

#1	‘Randomized Controlled Trials’ OR ‘Random allocation’ OR ‘Controlled Clinical Trials’ OR ‘Control groups’ OR ‘Clinical trials’ OR ‘Clinical Trials Data Monitoring Committees’ OR ‘Double- blind method’ OR ‘Single- blind method’
#2	‘Exercise’ OR ‘Exercise Therapy’ OR ‘Exercise’ OR ‘Physical Activity’ OR ‘Aerobic Exercise’ OR ‘Train’ OR ‘High- Intensity Interval Training’ OR ‘Cardiac Rehabilitation’ OR ‘respiratory muscle training’ OR ‘inspiratory training’ OR ‘exertion’. OR ‘Cardiovascular training OR ‘Endurance training’ OR ‘Tai Chi’ OR ‘Yoga’
#3	‘Sudden Cardiac Death’ OR ‘Cardiac Death, Sudden’ OR ‘Death, Sudden Cardiac’
#4	#1 and #2 and #3

### 2.5 Selection of studies

**Figure 1:**
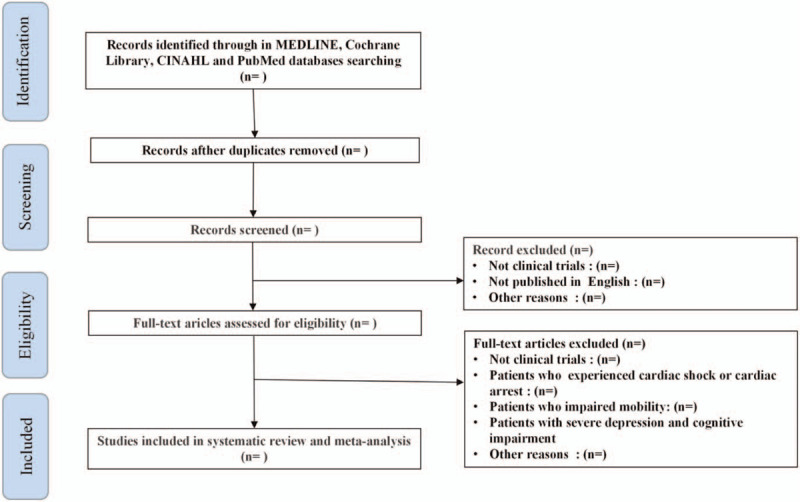
Flow chart of literature screening in this study.

All records will be imported into Endnote X9.3.3 (Clarivate Analytics, Philadelphia, Pennsylvania, USA) for document management; duplicate publications will be eliminated. After literature evaluation and ensuring the consistency of methodology, the same two reviewers will screen all titles and abstracts to determine the studies that meet the inclusion criteria. The full text of all shortlisted studies will be examined and reasons for exclusion will be provided, as required. The reference lists of all eligible studies will be screened for other potentially eligible studies. Study corresponding authors will be contacted by email in cases where article full text is not accessible or additional information about the study is required. Studies will be excluded if the required information is not obtainable. Any between-reviewer discrepancies at this stage will be resolved by consensus or third reviewer arbitration (Fig. 1).

### 2.6 Data collection and management

Standardized tables will be used for data extraction. The following data will be extracted:

1. Study information: author name, publication year, article title, journal title, research question and purpose, eligibility criteria, study design (cluster vs. non-cluster, single-center vs. multi-center), sample size, and follow-up duration;
2. Participant information: age, sex, ethnicity, weight, height, disease history;
3. Intervention: setting (community, rehabilitation center, hospital, home), classification, and exercise type (aerobic vs. resistance);
4. Outcomes: death rate; cause of death; hospitalization rate; duration, frequency, and intensity of exercise; observed exercise type (relative to the prescribed exercise type); energy consumption; reported adverse events; health-related quality of life; exercise compliance rates.

Two reviewers will calibrate the dataset by independently extracting randomly selected 30% of the included data. If the data extraction consistency is of ≥ 80% of the original consistency, the rest of the research data extraction will be extracted by one reviewer and examined by the other reviewer. If the original consistency rate is low, the data will be extracted independently by two reviewers and checked by a third reviewer. Any discrepancies will be resolved by consensus between two reviewers.

### 2.7 Study quality assessment

The evidence will be assessed independently by two reviewers, using the Grading of Recommendations Assessment, Development, and Evaluation system.^[22]^ The overall risk of bias will be evaluated, according to the Cochrane Handbook and the levels of evidence presented therein: high, medium, low, and very low. Randomized controlled trials are considered high-quality evidence. Nevertheless, several study-related factors will be evaluated, including the risk of bias (including publication bias), imprecision, inconsistency, indirectness, all of which may reduce the quality of evidence.

### 2.8 Risk of bias

The risk of bias and methodological quality of each study will be assessed by two reviewers, using the updated Cochrane Risk of Bias 2.0 tool.^[23]^ The following parameters will be evaluated: sequence generation, allocation concealment, blinding of participants and personnel, blinding of outcome evaluators, incomplete outcome data, and selective result reporting, among others. Any between-reviewer discrepancies will be resolved by consensus with the third reviewer. A bias risk chart will be generated to summarize the risk of bias assessment results.

### 2.9 Assessment of heterogeneity

The heterogeneity of the results will be evaluated by the I^2^ statistic. I^2^ values of ≥ 50% indicate high heterogeneity. In cases of high heterogeneity, sensitivity analysis will be performed to examine the sources of heterogeneity. In addition, homogeneity tests will be performed. Differences in study characteristics that may affect heterogeneity levels will be presented in a narrative synthesis.

### 2.10 Data synthesis

For dichotomous outcomes (such as adverse events, death), in the absence of hazard ratios, we will extract the number of participants who experienced the outcome of interest and the number of participants in each treatment group assessed at the end of the study to estimate a risk ratio. For continuous variables (for example, peak VO_2_, peak WR, VE/VCO_2_, LVEF, and 6MWD, levels, and quality of life, anxiety, and depression scores), the standardized mean difference (SMD) and the corresponding 95% confidence interval will be calculated. For studies that have used the same units of measurement, the average difference will be estimated as a therapeutic effect, or SMD will be used. If suitable, a meta-analysis of the review results will be performed. If I^2^ value is of ≥ 50%, a random effects model will be used for meta-analysis; otherwise, a fixed effect model will be used.

### 2.11 Subgroup analysis

Subgroup analyses will be performed if among-study heterogeneity is high or if a large number of trials is included. The following factors will be used for subgroup analyses: age (older adults vs. non-older adults), sex (male vs. female), weight (underweight, normal, overweight, obesity), comorbidities (hypertension, diabetes, CVD, and cerebrovascular diseases), types of exercise (aerobic vs. resistance exercise), frequency of exercise (< 3 times/week vs. ≥ 3 times/week), duration of exercise (< 12 weeks vs. ≥ 12 weeks), and intensity of exercise (low, medium, and high intensity).

### 2.12 Sensitivity analysis

After excluding low-quality studies, we will perform sensitivity analyses. One of the result indicators included in the study will be removed one by one to observe whether the heterogeneity has changed. If founding that there is a significant change in the heterogeneity of the resulting indicators after the removal of the literature, the literature is the source of heterogeneity. Consistency of study findings before and after sensitivity analyses suggests the robustness of the findings; in contrast, inconsistent results suggest that the study findings should be considered with caution.

## 3 Discussion

Physical activity is associated with risk. Previous studies have shown that sudden strenuous exercise increases the risk of SCD. Although the incidence of such events is low, the associated risk remains a concern for both doctors and patients. This study aims to clarify the relationship between the levels and types of exercise and SCD risk, using a meta-analysis. The findings can inform preventive policies and interventions. This study has some limitations. First, only English language articles are eligible. Second, the incidence of SCD caused by exercise is low; thus, few cases are likely to be included. Despite these limitations, this evidence will be relevant to practitioners and policy makers.

## Author contributions

**Conceptualization:** Qinqin wu, Rui Zeng.

**Data curation:** Yu Jia.

**Writing – original draft:** Qinqin wu, Fanghui Li, Yi Liu.

**Writing – review & editing:** Rui Zeng.
